# Radiological Patterns of Uveal Melanoma Liver Metastases in Correlation to Genetic Status

**DOI:** 10.3390/cancers13215316

**Published:** 2021-10-22

**Authors:** Serdar Yavuzyigitoglu, Michael C. Y. Tang, Miguel Jansen, Kaspar W. Geul, Roy S. Dwarkasing, Jolanda Vaarwater, Wojtek Drabarek, Robert M. Verdijk, Dion Paridaens, Nicole C. Naus, Erwin Brosens, Annelies de Klein, Emine Kilic

**Affiliations:** 1 Department of Ophthalmology, Erasmus Medical Centre Rotterdam, 3015 GD Rotterdam, The Netherlands; s.yavuzyigitoglu.1@erasmusmc.nl (S.Y.); Michael_cytang@msn.com (M.C.Y.T.); miguel_jansen@hotmail.com (M.J.); j.vaarwater@erasmusmc.nl (J.V.); w.drabarek@erasmusmc.nl (W.D.); D.Paridaens@oogziekenhuis.nl (D.P.); n.naus@erasmusmc.nl (N.C.N.); 2 Department of Clinical Genetics, Erasmus Medical Centre Rotterdam, 3015 GD Rotterdam, The Netherlands; e.brosens@erasmusmc.nl (E.B.); a.deklein@erasmusmc.nl (A.d.K.); 3 Department of Internal Medicine, Sint Franciscus Gasthuis Rotterdam, 3045 PM Rotterdam, The Netherlands; kwgeul@gmail.com; 4 Department of Radiology, Erasmus Medical Centre Rotterdam, 3015 GD Rotterdam, The Netherlands; r.s.dwarkasing@erasmusmc.nl; 5 Department of Pathology, Erasmus Medical Centre Rotterdam, 3015 GD Rotterdam, The Netherlands; r.verdijk@erasmusmc.nl; 6 The Rotterdam Eye Hospital, 3011 BH Rotterdam, The Netherlands

**Keywords:** uveal melanoma, choroidal melanoma, eye melanoma, BAP1, SF3B1, liver metastases

## Abstract

**Simple Summary:**

Patients with uveal melanoma develop metastases that almost always affect the liver. These liver metastases can have as different metastatic patterns. In this study we investigated the role of the mutation status of the primary tumor in this metastatic process. Mutations in *BAP1* or *SF3B1* did not correlate with a specific hepatic metastatic pattern, whereas chromosome 1p loss and 8p loss were much more frequent in the primary uveal melanomas of patients who eventually develop miliary metastases in comparison to patients who develop single solitary hepatic metastases. Future endeavors could focus on discovering additional (genetic) factors which influence the propagation and development of hepatic metastases in UM.

**Abstract:**

This study reports the role played by the mutation status of Uveal Melanoma (UM) in relation to hepatic metastatic patterns as seen on imaging modalities. Radiological images were obtained from 123 patients treated at the Erasmus Medical Center Rotterdam or the Rotterdam Eye Hospital. Radiological images were derived from either computed tomography or magnetic resonance imaging. Hepatic metastatic patterns were classified by counting the number of metastases found in the liver. Miliary metastatic pattern (innumerable small metastases in the entire liver) was analyzed separately. Mutation status was determined in 85 patients. Median disease-free survival (DFS) and survival with metastases differed significantly between each of the metastatic patterns (respectively, *p* = 0.009, *p* < 0.001), both in favor of patients with less hepatic metastases. The mutation status of the primary tumor was not correlated with any hepatic tumor profiles (*p* = 0.296). Of the patients who had a solitary metastasis (*n* = 18), 11 originated from a primary *BAP1*-mutated tumors and one from a primary *SF3B1*-mutated tumor. Of the patients who had a miliary metastasis pattern (*n* = 24), 17 had a primary *BAP1*-mutated tumor and two had a primary *SF3B1*-mutated tumor. Chromosome 8p loss was significantly more in patients with more metastases (*p* = 0.045). Moreover, the primary UMs of patients with miliary metastases harbored more chromosome 8p and 1p loss, compared to patients with single solitary metastasis (*p* = 0.035 and *p* = 0.026, respectively). In conclusion, our study shows that there is an inverse correlation of the number of metastasis with the DFS and metastasized survival, indicating separate growth patterns. We also revealed that the number and type of metastases is irrelevant to the prognostic mutation status of the tumor, showing that both *BAP1*- and *SF3B1*-mutated UM can result in solitary and miliary metastases, indicating that other processes lay ground to the different metastatic patterns.

## 1. Introduction

Uveal Melanoma (UM) is the most common primary intra-ocular malignancy in the Western World with an incidence of 5–6 per million per year [1,2]. UM derive from the melanocytes in the uveal tract which consists of the choroid, ciliary body and iris [3]. Treatment of the UM consists of external beam radiotherapy, brachytherapy or enucleation. However in spite of excellent local tumor control, UM has a strong propensity to metastasize in up to 50% of the patients within 15 years after diagnosis [4]. Localization of these metastases can occur in the liver, pulmonary parenchyma, bone and skin, among other sites. However, the liver is affected in more than 90% of the patients [4].

UM are characterized by nonrandom cytogenetic aberrations and recurrent mutated genes that are associated with the patients’ prognosis. The onset of metastatic UM is closely related to the presence of somatic mutations in the UM genes: *BAP1*, *SF3B1* and *EIF1AX* [5]. *BAP1*-mutated UM being classified as high risk with early metastases, *SF3B1*-mutated UM as intermediate risk with late-onset metastases and *EIF1AX*-mutated UM as low risk for metastases [5]. As for chromosomal changes, loss of chromosome 3 is most strongly correlated to metastatic disease together with the gain of chromosome 8q [6,7]. Since the *BAP1* gene is located on chromosome 3, the double hit of loss of chromosome 3 with a *BAP1* mutation on the remaining allele, results in the loss of BAP1 expression [8]. Other chromosomal aberrations that are associated with the worse prognosis include loss of chromosome 1p, 6q and 8p. Chromosome 6p gain is associated with a favorable prognosis [6,7].

The genetics of primary UM have been investigated intensively, however not much is known about the UM metastases (UM^meta^). The liver is typically affected in metastasized UM; thus, biopsies are not taken regularly to confirm the diagnosis of metastasis since findings on Computed Tomography (CT) scans and Magnetic Resonance Imaging (MRI) techniques suffice to make the diagnosis. Consequently, obtaining metastasis material is rare in UM research. Nevertheless, several studies have been performed in which UM^meta^ are investigated.

Meir and colleagues investigated the gene expression of primary UM compared to matching metastases and showed that the expression of 193 genes differed between the primary and metastasized UM [9]. Moreover, the gene expression of the liver metastasis resembled normal liver tissue, whereas it was histopathologically shown that the investigated metastasis material did not contain normal liver tissue [9]. Looking at chromosomal aberrations in UM^meta^, Trolet and colleagues showed that the majority of UM^meta^ harbor monosomy 3 with chromosome 8q gain [7]. However a smaller proportion of these tumors are disomy 3 with a gain of the terminal end of chromosome 8q [7]. It is very likely that these UM^meta^ contain *SF3B1* mutations, since disomy 3 with gain of the terminal end of chromosome 8q is a characteristic for *SF3B1*-mutated UM [10]. *SF3B1* mutations in UM^meta^ were described in 40% in one study (2/5 UM^meta^) and 4% in another study (1/26 UM^meta^) [11,12]. Loss of BAP1 expression and *BAP1* mutations were also shown to be frequently present in the majority of UM^meta^ [11,12,13]. More recently, Shain and colleagues investigated genetic alterations in matched primary tumor with metastases [14]. This revealed that almost all known driver mutations were identical in the matched tissue, however did reveal additional genetic alteration in the metastases that suggests oncogenic evolution after dissemination, which was also confirmed in other studies [14,15,16].

For other malignancies, such as cutaneous melanoma, breast cancer and colorectal carcinomas, it has been shown that the liver metastases display different growth pattern that are correlated to the prognosis [17,18,19]. Hepatic metastases in patients with UM also vary in size and pattern, from solitary lesions to a more disseminated or miliary pattern [20]. Since earlier research has shown that the genetic status of the primary UM can affect the risk for metastatic disease, we hypothesize that different genetic aberrations in the primary tumor will correlate to different hepatic metastases patterns. In this current study we set out to analyze the difference in survival for the metastasis patterns and also correlate these to clinical, histopathological and genetic parameters of the primary UM.

## 2. Materials and Methods

### 2.1. Patient Inclusion

All patients with UM who were diagnosed with UM between 1993 and 2021 at the Erasmus University Medical Center and the Rotterdam Eye Hospital (Rotterdam, The Netherlands) were reviewed for metastatic disease. Iris melanoma were not included due to their aberrant metastatic behavior and different genetic composition [15,21]. Following, we selected patients for which CT or MRI images of the metastatic liver were available for analyses. This study was performed according to the tenets of the Declaration of Helsinki and an informed consent was obtained before intervention. The study was also approved by a local ethics committee (reference number MEC-2014-627).

### 2.2. Analyses of Metastasis Imaging

Classification of CT or MRI images were based on the total amount of hepatic metastases. For statistical reasons, we chose not to use the number of lesions as a continuous variable, since this would skew the mean and median towards a high count due to the effect of a miliary metastatic pattern (innumerable small metastases). Solitary lesions, and more than 10 lesions throughout the liver were two obvious groups to make. For patients with 2 to 10 hepatic lesions, we chose to divide this in two groups to gain more statistical power for the analyses despite limited cases. This resulted in the following four groups: single nodular lesion, between two and five lesions, between 6 and 10 lesions and more than 10 lesions. The first known and available CT or MRI images that confirmed hepatic metastases were used for analysis. Measurements of the CT or MRI images were taken manually with built-in measuring tools to determine the dimensions of hepatic metastases. For this current paper we did not analyze the hepatic tumor burden (size of the independent hepatic lesions) since the aim of this study was to correlate the pattern of hepatic metastases to the different clinical, histopathological and genetic parameters.

### 2.3. Material and Genetic Analyses

Whenever a patient was enucleated or a biopsy was taken, tumor material was available for genetic analyses. This was also the case for patients who underwent secondary enucleation or endoresection of the tumor, The genetic data used in this paper is thoroughly described our previous publications [5,8,22]. In short, the mutation status was determined using a combination of BAP1 immunohistochemistry (IHC), Sanger sequencing or sequencing using the ION Torrent Personal Genome Machine (Life Technologies, Carlsbad, CA, USA) as described before [22]. The genetic status was defined as either BAP1 aberrant, *SF3B1*-mutated, or No Recurrent Mutations (NRM). NRM tumors were BAP1 IHC positive or/and *BAP1* wildtype with also an *SF3B1* wildtype mutation status. Chromosome analyses were conducted using either single nucleated polymorphism array analysis (HumanCytoSNP-12 version 2.1 BeadChip and Illumina 610Q BeadChip; Illumina, San Diego, CA, USA), or fluorescence in situ hybridization as described before [22].

### 2.4. Statistical Analysis

The disease-free survival (DFS) is defined as the first date of treatment until the detection of metastatic UM. Survival with metastasis was calculated using the first confirmed date of metastatic UM until death or the last follow-up date. Deaths due to other causes were censored. The survival analysis was generated using the Kaplan-Meier method and made use of the log-rank test to find differences between the groups. Pearson’s chi-square test or Fisher’s exact test was used to test categorical groups with independent variables. Analysis of variance (ANOVA) was used to compare the means of multiple groups, while Fisher’s least significant difference and Tukey method were used for post hoc testing. *p* values of 0.05 or less were considered significant. SPSS (version 24.0, IBM, Armonk, NY, USA) was used for all statistical analyses.

## 3. Results

A total of 234 patients with UM^meta^ were identified. The location of metastasis was not recorded for 51 patients. These patients received their follow-up elsewhere and only the presence of metastases, and subsequently the survival, was shared by the general practitioner. For the remaining 183 patients the location of the metastasis was known. The liver was affected in 175 patients (95.6%) and extra-hepatic metastases were found in 70 patients (39.3%); in 62 patients this was in addition to the hepatic metastasis and in 10 patients metastases occurred without hepatic UM metastases. However, two of these latter patients still developed hepatic metastases after initially only the diagnosis of extra-hepatic metastases. CT or MRI images of the liver metastases were available for 123 patients. This was the eventual cohort used for analyses.

From the included patients (*n* = 123) a total of 77 underwent primary enucleation and 43 received stereotactic radiation therapy (SRT) of which five underwent secondary enucleation (four due to local progression; one due to neovascular glaucoma), and four underwent endoresection of the tumor (due to toxic tumor syndrome). One patient was treated with brachytherapy and underwent enucleation due to local progression. Two patients did not receive primary treatment. These patients were diagnosed with metastasis at the time of diagnosis and wished not to receive treatment of the primary tumor. Clinical, histopathological and genetic characteristics of the study population are summarized in Table 1. The study population comprised of 57 males and 66 females with a median age of 60 years at diagnosis of the primary UM (range, 28–87 years). The median DFS was 25.6 months (range 0–159.6 months) and median survival with metastatic disease was 8.0 months (range, 0–114.3 months). Mutation status of the primary UM was known for 85 patients; 73 with aberrant BAP1 (either BAP1 IHC negative or *BAP1* mutation, depending on the available tests), seven with an *SF3B1* mutation and five without an aberrant BAP1 or an *SF3B1* mutation (No Recurrent Mutation). In 25 patients there was a hotspot mutation in *GNAQ*, and for 29 patients in *GNA11*. UM tumors of five patients were wildtype for both genes. In four of these patients we sequenced the hotspot location of *PLCB4* and *CYSLTR2*. In two patients the tumor harbored a *PLCB4* p.Asp630Phe mutation, in one patient there as a *CYSLTR2* p.Leu129Gln mutations, and one patient was wildtype for these two genes as well. The latter case, and the other *GNAQ*/*GNA11* wildtype tumor, did both show a high frequency of monosomy 3, concluding that the sequenced material was indeed tumor material. For 38 patients the mutation status was unknown, and patients treated with SRT amassed the greatest share with 34 of the 38 unknown mutations pattern, due to lack of material of the primary tumor. An overview of all patients with details of primary treatment, number of metastases, gene status and chromosomal abnormalities are displayed in Figure S1.

### 3.1. Survival Analysis

**Figure 1:**
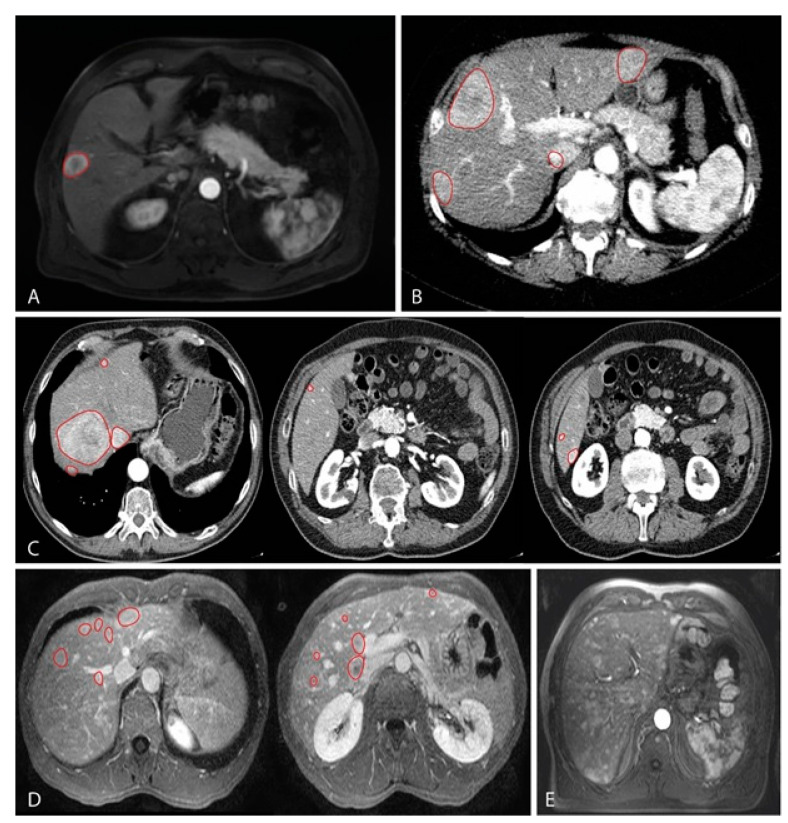
MRI images representing the classification based on the number of lesions found in metastatic UM in the liver, which have been contrasted by red markings; (**A**) T1 weighted MRI with contrast showing a single nodular lesion in the liver; (**B**) T2 weighted MRI with contrast showing four lesions; (**C**) T2 weighted MRI sequence with contrast showing a total of seven lesions. Images from left to right correspond, respectively, from cranial to caudal localization; (**D**) T2 weighted MRI sequence with contrast showing a total of 12 lesions. Images from left to right correspond, respectively, from cranial to caudal localization; (**E**) T2 weighted MRI with contrast showing innumerable widespread small lesions throughout all liver segments. No red markings are present due to the number of lesions.

Classification of CT or MRI images were based on the total amount of hepatic metastases, this resulted in the following groups: single nodular lesion (Figure 1A), between 2 and 5 lesions (Figure 1B), between 6 and 10 lesions (Figure 1C) and more than 10 lesions (Figure 1D). Survival analyses were performed by using at the time from diagnosis up until metastatic disease (DFS) and survival with metastatic disease.

For these analyses we only used the number of metastases that were present at the initial CT or MRI scan when the first metastases were observed. As such, 18 patients (14.6%) presented with one solitary metastasis. There were 35 patients (28.5%) who presented with 2 to 5 liver metastatic lesions. In addition, there were 17 patients (13.8%) with 6 to 10 hepatic metastases. Strikingly, 53 patients (43.1%) presented with more than 10 liver metastases.

**Figure 2:**
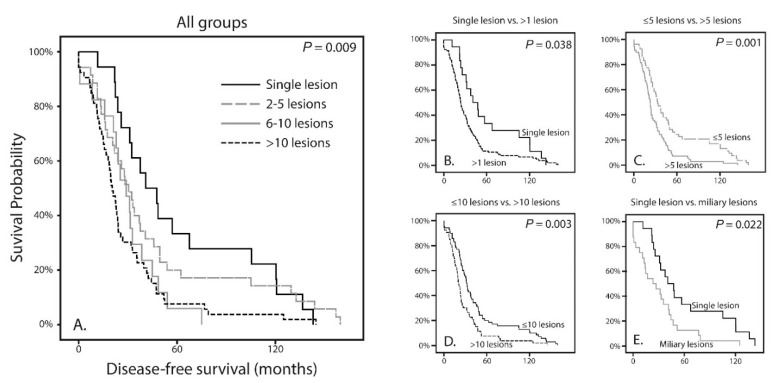
Kaplan-Meier curves showing the disease-free survival of the four classified tumor profiles as well as of multiple comparisons; (**A**) The DFS differs significantly between the groups (*p* = 0.009); (**B**) The DFS is significantly more favorable in patients who have a single hepatic lesion compared to patients with more than one lesion (*p* = 0.038); (**C**) The DFS is significantly more favorable in patients who have less than five hepatic lesion compared to patients with more than five lesion (*p* = 0.001); (**D**) The DFS is significantly more favorable in patients who have less than 10 hepatic lesion compared to patients with more than 10 lesion (*p* = 0.001); (**E**) Comparison of patients with a single hepatic lesion with patients who have a miliary hepatic metastatic pattern reveals a worse DFS in the miliary group (*p* = 0.022).

**Figure 3:**
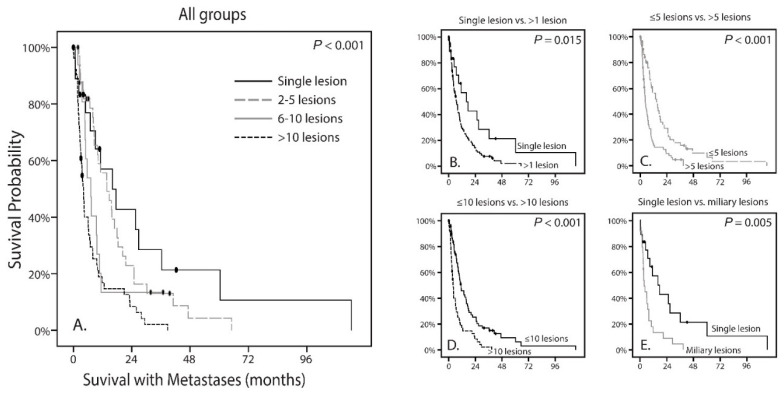
Kaplan-Meier curves showing the survival with metastases of the four classified tumor profiles as well as of multiple comparisons; (**A**) The survival differs significantly between the groups (*p* < 0.001); (**B**) The survival is significantly more favorable in patients who have a single hepatic lesion compared to patients with more than one lesion (*p* = 0.015); (**C**) The survival is significantly more favorable in patients who have less than five hepatic lesion compared to patients with more than five lesion (*p* < 0.001); (**D**) The survival is significantly more favorable in patients who have less than 10 hepatic lesion compared to patients with more than 10 lesion (*p* < 0.001); (**E**) Comparison of patients with a single hepatic lesion with patients who have a miliary hepatic metastatic pattern reveals a worse DFS in the miliary group (*p* = 0.005).

We observed a difference for DFS when comparing all groups (*p* = 0.009; Figure 2A). This showed a shorter DFS with increasing number of hepatic metastases. We also analyzed using thresholds rather than separate groups. These were set on one solitary lesion versus more than one (1 vs. >1); five or less versus more than five (≤5 vs. >5); 10 or less versus more than 10 lesions (≤10 vs. >10). Each time the DFS was worse in patients with more metastases (*p* = 0.038, *p* = 0.001 and *p* = 0.003, respectively; Figure 3B, Figure 3c and Figure 3D). Histopathologically there are distinctive liver growth patterns in metastasized UM; the nodular and infiltrative growth patterns [23,24]. The infiltrative growth pattern is characterized by many metastases that are up to 50 µm large [23,24]. This pattern of innumerable small metastasis is also named miliary pattern on CT and MRI images. Upon reviewing CT and MRI images of the >10 lesions group (*n* = 53), we divided this group into two separate groups. A group (*n* = 29) containing samples with more than 10 lesions (larger than 50 µm; Figure 1D) and a group (*n* = 24) containing samples with a miliary pattern (innumerable lesions smaller than 50 µm; Figure 1E). Comparing both groups did not show a significant difference in DFS (*p* = 0.375).

Following, we analyzed whether the number of metastases in the liver were correlated to the survival with metastases. A difference was observed when comparing all groups (*p* < 0.001; Figure 3A). Using the same thresholds as described before, there was a significant difference in survival with metastases for patients between one lesion compared to more than one lesion (*p* = 0.015; Figure 3B). This significant difference was also observed for ≤5 versus >5 lesions (*p* < 0.001) and ≤10 versus >10 lesions (*p* < 0.001), all in favor of the lesser number of metastases (Figure 3C,D). In addition, similar to DFS, no difference was observed between more than 10 lesions and a miliary pattern (*p* = 0.638).

Furthermore, we analyzed whether the presence of extra-hepatic metastases would be correlated to either the overall survival of patients or the amount the hepatic metastases. In this cohort of 123 patients, 50 patients also had extra-hepatic metastases. Patients with extra-hepatic metastases did not differ in time until first metastasis (*p* = 0.213) compared to patient with only hepatic metastatic disease. In addition, the survival with metastases did not differ if extra-hepatic metastases were present (*p* = 0.399). In patients with one solitary hepatic metastasis 56% (*n* = 10/18) also had extra hepatic metastases. In patients with 2 to 5 hepatic metastases, extra-hepatic metastases were present in 37% (*n* = 13/35). For patients with 6 to 10 hepatic metastases this was present in 29% (*n* = 5/17) and in patients with more than 10 metastases extra-hepatic metastases were present in 42% (*n* = 22/53). This was overall not significantly different between the patient groups (*p* = 0.433, Table 2). In half of the patients with miliary metastatic pattern (*n* = 12/24), there were also extra-hepatic metastases present.

### 3.2. Correlation with Clinical, Histopathological and Genetic Features of the Primary Tumor

**Figure 4:**
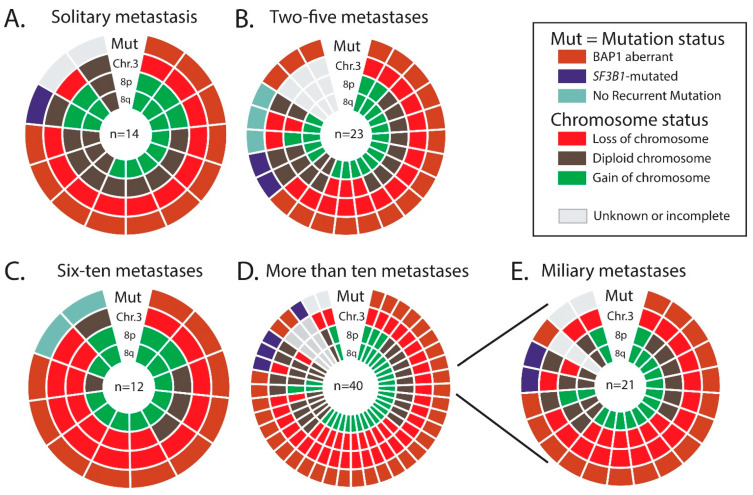
A doughnut chart with the mutation status of the prognostic relevant genes (*BAP1*, *SF3B1* and No Recurrent Mutation), and aberrations of chromosome 3, 8p and 8q in the primary uveal melanomas, for respectively patients with (**A**) solitary metastases, (**B**) 2 to 5 metastases, (**C**) 6 to 10 metastases and (**D**) more than 10 metastases. (**D**,**E**) The distribution of the genetic abnormalities in patients who developed miliary metastases.

The age at diagnoses of the primary UM was not significantly different between the patients (*p* = 0.599). In addition, gender was equally divided between the groups (*p* = 0.801). A trend could be observed for largest basal diameter of the primary UM, for which a larger diameter of the primary UM was to give more metastases, however no significance could be reached (*p* = 0.072). Tumor height also did not differ between the metastasis groups (*p* = 0.345). Histopathological features in the primary UM such as the presence of epithelioid cell type, presence of closed vascular loops, involvement of the ciliary body and extra-ocular extensions were all shown not be associated with the number of metastases (Table 2). BAP1 IHC, *SF3B1*, *EIF1AX*, *GNAQ* and *GNA11* mutation status were not found to be significantly associated with the number of metastases. (Table 2). Abnormalities of chromosome 1p, 3, 6p, 6q, and 8q were not associated with the number of metastases, however a significant difference (*p* = 0.045) was observed for chromosome 8p (Table 3). Even though this would not remain significant if we would correct for multiple testing, it is clear that loss of chromosome 8p is totally absent in the primary UMs of patients who have a single metastasis. An overview of the status of BAP1 and *SF3B1*, together with the status of chromosome 3, 8p and 8q is shown in Figure 4A–D, separated for the, respectively, number of metastases.

### 3.3. Differences between Solitary Metastases and Miliary Metastases Pattern

Earlier research has shown that radiological patterns are clearly distinct for two groups, in which a histological nodular growth pattern matches the solitary (large) metastasis and the histological infiltrative growth pattern matches the innumerable small metastases (miliary metastatic pattern) [25]. Since the solitary metastasis and the miliary metastases pattern are clearly distinct on the CT and MRI scans of the liver, and also seem to correlate with the different histopathological growth patterns, we set to analyze whether differences between clinical and genetic parameters would be more noticeable [25]. As mentioned before, the time until metastases (*p* = 0.022; Figure 2E) and the survival with metastases (*p* = 0.005; Figure 3E) are significantly worse for patients with a miliary metastases pattern and thus in favor of patients with solitary metastases. Within patients with either solitary or miliary hepatic metastases, the presence of extra-hepatic metastases did not significantly influence the survival with metastases (*p* = 0.410 and *p* = 0.852, respectively). Moreover, none of the clinical (age, gender, LTD, tumor thickness), histopathological (epithelioid cell type, closed vascular loops, involvement ciliary body and extra-ocular extensions) and genetic parameters (*BAP1*, *SF3B1*, *EIF1AX*, *GNAQ* and *GNA11*) were significantly different (all *p* values above 0.05). Of the patients who had a solitary metastasis (*n* = 18), 11 had an aberrant BAP1 tumor and one with an *SF3B1*-mutated tumor. In six patients the mutation status was unknown due to lack of tumor material. Of the patients who had a miliary metastasis pattern (*n* = 24), 17 had an aberrant BAP1 tumor and two had an *SF3B1*-mutated tumor. In five patients with miliary metastases the mutation status was unknown due to lack of tumor material. Chromosomal abnormalities were not significantly different for chromosome 3, 6p, 6q, and 8q. For chromosome 1p and chromosome 8p there was a significant difference observed (*p* = 0.026 and *p* = 0.035, respectively). Loss of chromosome 1p was present in only 21% (3/14) of the UMs of patients with solitary metastases, whereas chromosome 1p loss was observed in 60% (12/20) of the UMs in patients with miliary metastases (Table 3. Chromosome 8p loss was totally absent (0/14; 0%) in the solitary group, whereas it was present in 7/20 (35%) UMs with miliary metastases (Figure 4A,E). In addition, the gain of chromosome 8p (in the form of gain of entire chromosome 8) was more frequent in the solitary group (5/14; 36%) compared to the miliary group (3/20; 15%).

## 4. Discussion

UM is the most common primary intra-ocular malignancy of the adult eye with a strong propensity to metastasize to the liver. A great deal of progress has been made in finding prognostic genetic markers in UM, however less is known about the hepatic metastasis in UM. Even though a limited number of publications regarding this topic have appeared, none of them have focused specifically on the relation between the mutation status of the primary UM and the number or pattern of metastases.

In this current study we found that regardless of the mutation status of the primary UM, the metastases in these patients display the same metastatic profile. Patients with an aberrant BAP1 UM and patients with *SF3B1*-mutated UM, both develop solitary hepatic metastases and both develop a miliary hepatic metastases pattern. This concurs with another research, in which there was no association of chromosome 3 or chromosome 8q with the two different growth patterns of the metastases [26]. Patients with monosomy 3 with or without chromosome 8q gain in the UM, developed both types of metastatic pattern. These tumors were most likely BAP1 aberrant [10,27]. However also patients with disomy 3 with chromosome 8q gain, developed both types of metastatic pattern [26]. These disomy 3 with chromosome 8q gain tumors are most similar to *SF3B1*-mutated as we and others have shown before [10,27]. *BAP1*, a tumor suppressor gene, is involved in DNA-repair, most notably double strand breaks and *SF3B1*, a splicing factor, is involved in alternative splicing of other genes [28,29]. While both genes play a different role in the tumorigenesis, it is very interesting that both result in different metastatic patterns.

Strikingly, we did find a significant difference in chromosome 8p loss, which was more frequent in the primary UMs of patients with more metastases. Moreover, this was even more noticeable when we compared the single solitary group with the miliary group, where chromosome 8p loss was absent in the UMs of patients with a solitary metastasis. Another study also found a correlation of chromosome 8p loss with the infiltrative hepatic growth patterns, which would coincide with a miliary pattern [30]. Since also chromosome 1p loss was observed almost three times more often in the UMs of patients with miliary metastases, compared to single metastases, we hypothesize that these differences could be the product of isochromosome formation. Our previous research has shown that more copies of chromosome 8q (with subsequent 8p loss) is associated with a more aggressive disease [31]. However, it is also suggested that metastatic growth properties are modulated by suppression of gene regions specific to chromosome 8p, irrespectively of chromosome 8q gain [32]. This not only underlines the importance of genes localized on chromosome 8p for the spread and development of hepatic metastases in patients with UM, but it is also indicative for the role played by the genetic landscape of the primary tumor in the homing of tumor cells.

Interestingly enough, the DFS differed between the groups. The single nodular lesion group showed a longer DFS compared to groups with more lesions. If the assumption was made that hepatic metastasis grows in one linear or exponential fashion, single nodular UM^meta^ should have a shorter DFS than UM^meta^ with multiple lesions, by hypothesizing that if a patient is diagnosed with one lesion it is earlier in the same metastatic process than a patient who is diagnosed with multiple metastases. However, this was not the case, making it very likely that UM^meta^ with single nodular lesions have a slower growth and are distinct from UM^meta^ with multiple lesions. Histopathological findings in UM^meta^ showed that these tumors indeed exhibit two types of growth patterns in the liver [23,24]. An infiltrative growth pattern which usually presents as many small (size < 50 µm) lesions, also named lobular or replacement pattern. The other growth pattern is the nodular growth, which contain less but larger lesions (>50 µm) [24]. This nodular growth is also described as portal, desmoplastic and pushing pattern. Both growth patterns have distinct anatomical locations, and also mixtures of both growth patterns in one affected liver are described [24,26,33]. The difference in DFS is also shown for the different growth patterns, in which the mean average doubling time of the infiltrative UM metastasis is significantly less than that of the nodular UM metastasis [25]. Our current study is limited since we only take the number of lesions into count, making it likely that especially the UM^meta^ with more than 10 lesion contain metastases with a pushing and replacement growth patterns. To compensate for this, we divided the UM^meta^ with more than 10 lesions into two groups; more than 10 large (>50 µm) lesions and the miliary pattern (<50 µm). This did not show a difference between these two groups; however, a clear difference can be seen between UM^meta^ with single lesions and miliary lesions. Ideally, we would cross reference with histopathology to confirm specific nodular, miliary or mixed patterns. However, despite these limitations we were still able to distinguish nodular and infiltrative growth based on radiological imaging without resorting to histopathology.

We hypothesized that the different types of lesions would correspond to the mutation status of the primary UM since we previously reported that *BAP1*-mutated UMs display rapid metastases and *SF3B1*-mutated UMs display late-onset metastases [5]. Strikingly this was not the case. It is thus very interesting that, irrespective of the mutation status, UM^meta^ with a *BAP1* mutation or *SF3B1* mutation can show both growth patterns. This might explain why a small part of the patients with *SF3B1*-mutated UMs have early-onset metastasis and *BAP1*-mutated UM have late-onset metastasis. It remains debatable whether this metastatic spread is purely by chance or whether there is a different or additional genetic predisposition of the primary UM that is not yet discovered. It has been shown that UM^meta^ have additional mutations which are exclusive to the metastases [14]. Furthermore, it is also shown that UM with a gene expression profile (GEP) Class 1 metastasize more often and more frequently extra-hepatic (50%) compared to GEP Class 2 UMs (10%) [34]. This not only underlines the importance of genetics for the spread and development of hepatic metastasis in UM, but it is indicative for the role played by the genetic landscape of the primary tumor in the homing of tumor cells. An alternative explanation for the differences in UM metastatic patterns in the liver might lie in a variation of expression of integrin α2 adhesive molecules. These molecules have been associated with the selective potential of UM for the formation of hepatic metastasis [35].

Another discovery was the amount of extra-hepatic metastases in patients with UM. Approximately 40% of the patients develop metastases outside the liver, and in most cases with the presence of hepatic metastases. Strikingly, this did not significantly influence the survival, probably since hepatic metastases are almost always lethal with the lack of a successful life-enhancing treatment in this historical cohort. We are currently investigating the role of the genetics of the primary tumor with the different locations of metastases.

Research into UM^meta^ is not only challenging due to the lack of material, but also due to the bias which is created by the methods used to obtain metastasis material. Material is usually available through hepatectomy and this is only performed in patients who are suited for this therapy and not for patient with a miliary pattern. However, research into the genetics of metastases remains important, especially since there are several reports which state the different metastatic patterns also react differently to therapy [30,33,36]. It has been shown that patients with a nodular metastatic growth pattern whom receive hepatic arterial chemoembolization have a significant longer survival [30,36]. One report even shows that in a patient with a mixture of infiltrative and nodular metastatic growth pattern, the nodular metastasis show reduction in size to hepatic radio-embolization whereas the infiltrative metastasis remain resistant and persist in growth [33]. This highlights that targeted therapy and perhaps also combination therapy is the future in UM^meta^ therapy. Patients could be treated with targeted precision whenever the UM^meta^ would present with a characteristic metastasis pattern.

A major limitation of this study was the lack of histopathological confirmation of the different metastatic growth patterns. Especially since these different growth patterns can occur in the same liver. However, by performing sub analyses where we only selected patients with one solitary metastasis and compared it to patients with a distinctive miliary pattern of hepatic metastases, we were able to exclude that one type of genetic mutation in the known UM genes was responsible for one type of metastatic growth pattern. For this study we did not use the hepatic tumor burden as a parameter and only took into count the number of metastases. Hepatic tumor burden has been shown before to be a prognostic marker in patients with UM^meta^ [37]. However, for this study the main aim was to investigate whether the different hepatic metastasis patterns (e.g., single solitary versus miliary pattern) was associated with the known clinical, histopathological and genetic features of the patient and their primary UM.

## 5. Conclusions

Our study has shown that there is an inverse correlation of the number of metastasis with the metastasized survival, indicating separate growth patterns. We could not find an association of metastasis with the mutation status, however did find that chromosome 1p and 8p loss were much more frequent in the UMs of patients who have miliary metastases compared to patients with single solitary metastasis. Future endeavors could focus on discovering additional (genetic) factors which influence the propagation and development of hepatic metastases in UM.

## Figures and Tables

**Table 1:** Clinical and genetic features of the cohort (*n* = 123).

Variables	Total
	Mean (range)
Age (years)	60.3 (28–87)
Largest basal diameter (mm)	13.7 (5–20)
Tumor thickness (mm)	7.0 (1–22)
Gender	*n* (%)
Male	57/123 (46%)
Female	66/123 (54%)
Survival	Median (range)
Disease-free survival, months	25.6 (0–159.6)
Survival with metastases, months	8.0 (0–114.3)
	No (%)
Alive	17/123 (14%)
Death due to metastasis	106/123 (86%)
Death due to other cause	0/123 (0%)
Primary treatment UM	*n* (%)
Enucleation	77/123 (63%)
Irradiation	44/123 (36%)
Irradiation with secondary treatment	10/44 (23%)
No treatment	2/123 (2%)
Mutation primary UM	*n* (%)
BAP1 aberrant	73/85 (86%)
SF3B1-mutated	7/85 (8%)
No Recurrent Mutation	5/85 (6%)
Hepatic metastatic patterns	*n* (%)
Single lesion	18/123 (14.6%)
2 to 5 lesions	35/123 (28.5%)
6 to 10 lesions	17/123 (13.8%)
More than 10 lesions	53/123 (43.1%)

**Table 2:** Correlation between the number of metastatic lesions and Other Clinical, Histopathological, and Genetic Features.

Variables	Hepatic Metastatic Patterns (Number of Metastases)				
	1 Lesion	2–5 Lesions	6–10 Lesions	>10 Lesions	*p*-Value
Mean age (range), yrs	59.9 (39–81)	58.4 (28–87)	62.0 (44–80)	61.2 (37–83)	0.599 *
Mean largest basal diameter UM (range), mm	13.2 (9–17)	12.7 (10–18)	13.9 (9–19)	14.3 (5–20)	0.072 *
Mean tumor height UM (range), mm	6.2 (2–10)	6.5 (2–12)	7.0 (1–13)	7.8 (2–22)	0.345 *
Gender (%), *n*					
Male	7 (39%)	15 (43%)	8 (47%)	27 (51%)	0.795†
Female	11 (61%)	20 (57%)	9 (53%)	26 (49)	
Histopathological features primary UM, *n*					
Epitheliod cells	11/13	22/24	12/12	30/37	0.319 †
Closed vascular loops	6/11	10/24	8/11	23/35	0.211 †
Involvement ciliary body	4/12	8/24	5/12	13/35	0.961 †
Extra-ocular extensions	0/11	2/23	4/12	7/31	0.095 †
Mutation primary UM, *n*					
BAP1 aberrant	11	18	10	34	0.296 †
*SF3B1*-mutated	1	2	0	4	
No Recurrent Mutation	0	3	2	0	
Not tested/incomplete	6	12	5	15	
Mutation primary UM, *n*					
*GNAQ*-mutated	2	9	5	9	0.414 †§
*GNA11*-mutated	6	6	4	13	0.502 †§
*GNAQ*/*GNA11* wildtype	2	2	0	1	
Not tested	8	18	8	30	
Extrahepatic metastases, *n*					
Yes	10	13	5	22	0.433 †
No	8	22	12	31	

* One-way analysis of variance (ANOVA). † Pearson’s chi-square test, § Tested as mutation vs. wildtype.

**Table 3:** Correlation between the number of metastatic lesions and the chromosomal abnormalities.

	Hepatic Metastatic Patterns (Number of Metastases)						
Variables (*n* = 83)	1 Lesion	2–5 Lesions	6–10 Lesions	>10 Lesions	*p*-Value	Miliary	*p*-Value *
Chromosome 1p							
Loss	3	7	6	17	0.352	12	0.026
Normal	11	13	6	20		8	
Chromosome 3							
Loss	12	17	11	33	0.932	18	0.703
Normal	2	3	1	4		2	
Chromosome 6p							
Loss	0	0	1	1	0.796	15	0.816
Normal	10	16	8	28		5	
Gain	4	4	3	8			
Chromosome 6q							
Loss	2	4	4	12	0.531	6	0.305
Normal	11	15	8	25		14	
Gain	1	1	0	0		0	
Chromosome 8p							
Loss	0	4	6	13	0.045	7	0.035
Normal	9	13	3	19		10	
Gain	5	3	3	5		3	
Chromosome 8q							
Normal	4	2	1	9	0.332	5	0.816
Gain	10	18	11	28		15	

All tests were tested using the Pearson’s chi-square test, * Pearson’s chi-square test between 1 lesion vs. military.

## Data Availability

The data presented in this study are available on request from the corresponding author.

## Supplementary Materials

The following are available online at https://www.mdpi.com/article/10.3390/cancers13215316/s1, Figure S1: A schematic overview of all patients with UM with the subsequent treatment, number of metastases, BAP1 IHC, mutations in *BAP1*/*SF3B1*/*EIF1AX*/*GNAQ*/*GNA11*, and abnormalities of chromosome 1p/3/6p/6q/8p/8q.

## List of Abbreviations

ANOVA	Analysis of variance
BAP1	BRCA1-associated Protein 1
CT	Computed Tomography
CYSLTR2	Cysteinyl Leukotriene Receptor 2
DFS	Disease-free survival
EIF1AX	Eukaryotic Translation Initiation Factor 1A, X-linked
GEP	Gene Expression Profile
GNA11	Guanine Nucleotide Binding Protein, subunit 11
GNAQ	Guanine Nucleotide Binding Protein, subunit q
IHC	Immunohistochemistry
MRI	Magnetic Resonance Imaging
NRM	No Recurrent Mutation
PLCB4	Phospholipase C Beta 4
SF3B1	Splicing Factor 3b subunit 1
UM	Uveal Melanoma
UM^meta^	Uveal Melanoma Metastases

## References

[1] Singh A.D., Turell M.E., Topham A.K. (2011). Uveal Melanoma: Trends in Incidence, Treatment, and Survival. Ophthalmology.

[2] Hu D.-N., Yu G.-P., McCormick S.A., Schneider S., Finger P.T. (2005). Population-Based Incidence of Uveal Melanoma in Various Races and Ethnic Groups. Am. J. Ophthalmol..

[3] McLaughlin C.C., Wu X.-C., Jemal A., Martin H.J., Roche L.M., Chen V.W. (2005). Incidence of noncutaneous melanomas in the U.S. Cancer.

[4] Kujala E., Ma¨kitie T., Kivelä T. (2003). Very Long-Term Prognosis of Patients with Malignant Uveal Melanoma. Investig. Opthalmol. Vis. Sci..

[5] Yavuzyigitoglu S., Koopmans A.E., Verdijk R.M., Vaarwater J., Eussen B., van Bodegom A., Paridaens D., Kilic E., de Klein A., Rotterdam Ocular Melanoma Study G. (2016). Uveal melanomas with sf3b1 mutations: A distinct subclass associated with late-onset metastases. Ophthalmology.

[6] Höglund M., Gisselsson D., Hansen G.B., White V.A., Säll T., Mitelman F., Horsman D. (2003). Dissecting karyotypic patterns in malignant melanomas: Temporal clustering of losses and gains in melanoma karyotypic evolution. Int. J. Cancer.

[7] Trolet J., Hupe P., Huon I., Lebigot I., Decraene C., Delattre O., Sastre-Garau X., Saule S., Thie´ry J.-P., Plancher C. (2009). Genomic Profiling and Identification of High-Risk Uveal Melanoma by Array CGH Analysis of Primary Tumors and Liver Metastases. Investig. Opthalmol. Vis. Sci..

[8] Koopmans A., Verdijk R.M., Brouwer R.W.W., Bosch T.V.D., Berg M.M.P.V.D., Vaarwater J., Kockx C., Paridaens D., Naus N.C., Nellist M. (2014). Clinical significance of immunohistochemistry for detection of BAP1 mutations in uveal melanoma. Mod. Pathol..

[9] Meir T., Dror R., Yu X., Qian J., Simon I., Pe’Er J., Chowers I. (2007). Molecular Characteristics of Liver Metastases from Uveal Melanoma. Investig. Opthalmol. Vis. Sci..

[10] Yavuzyigitoglu S., Drabarek W., Smit K.N., van Poppelen N., Koopmans A.E., Vaarwater J., Brands T., Eussen B., Dubbink H.J., van Riet J. (2017). Correlation of Gene Mutation Status with Copy Number Profile in Uveal Melanoma. Ophthalmology.

[11] Griewank K., Van De Nes J., Schilling B., Moll I., Sucker A., Kakavand H., E Haydu L., Asher M., Zimmer L., Hillen U. (2013). Genetic and clinico-pathologic analysis of metastatic uveal melanoma. Mod. Pathol..

[12] Luscan A., A Just P., Briand A., Roziers C.B.D., Goussard P., Nitschké P., Vidaud M., Avril M.F., Terris B., Pasmant E. (2014). Uveal melanoma hepatic metastases mutation spectrum analysis using targeted next-generation sequencing of 400 cancer genes. Br. J. Ophthalmol..

[13] McCarthy C., Kalirai H., Lake S.L., Dodson A., Damato B.E., Coupland S.E. (2015). Insights into Genetic Alterations of Liver Metastases from Uveal Melanoma. Pigment. Cell Melanoma Res..

[14] Shain A.H., Bagger M.M., Yu R., Chang D., Liu S., Vemula S., Weier J.F., Wadt K., Heegaard S., Bastian B.C. (2019). The genetic evolution of metastatic uveal melanoma. Nat. Genet..

[15] Karlsson J., Nilsson L.M., Mitra S., Alsén S., Shelke G.V., Sah V.R., Forsberg E.M.V., Stierner U., All-Eriksson C., Einarsdottir B. (2020). Molecular profiling of driver events in metastatic uveal melanoma. Nat. Commun..

[16] Durante M.A., Rodriguez D.A., Kurtenbach S., Kuznetsov J.N., Sanchez M.I., Decatur C.L., Snyder H., Feun L.G., Livingstone A.S., Harbour J.W. (2020). Single-cell analysis reveals new evolutionary complexity in uveal melanoma. Nat. Commun..

[17] Nielsen K., Rolff H.C., Eefsen R.L., Vainer B. (2014). The morphological growth patterns of colorectal liver metastases are prognostic for overall survival. Mod. Pathol..

[18] Barnhill R., Van Dam P., Vermeulen P., Champenois G., Nicolas A., Rawson R.V., Wilmott J., Thompson J., Long G., Cassoux N. (2020). Replacement and desmoplastic histopathological growth patterns in cutaneous melanoma liver metastases: Frequency, characteristics, and robust prognostic value. J. Pathol. Clin. Res..

[19] Frentzas S., Simoneau E., Bridgeman V.L., Vermeulen P.B., Foo S., Kostaras E., Nathan M.R., Wotherspoon A., Gao Z.-H., Shi Y. (2016). Vessel co-option mediates resistance to anti-angiogenic therapy in liver metastases. Nat. Med..

[20] Singh P., Singh A. (2012). Choroidal melanoma. Oman J. Ophthalmol..

[21] Henriquez F., Janssen C., Kemp E.G., Roberts F. (2007). The t1799a braf mutation is present in iris melanoma. Investig. Ophthalmol. Vis. Sci..

[22] Yavuzyigitoglu S., Mensink H.W., Smit K.N., Vaarwater J., Verdijk R.M., Beverloo B., Brüggenwirth H.T., Van Marion R., Dubbink H.J., Paridaens D. (2016). Metastatic Disease in Polyploid Uveal Melanoma Patients Is Associated With BAP1 Mutations. Investig. Opthalmol. Vis. Sci..

[23] Grossniklaus H.E. (2013). Progression of ocular melanoma metastasis to the liver: The 2012 zimmerman lecture. JAMA Ophthalmol..

[24] Grossniklaus H.E., Zhang Q., You S., McCarthy C., Heegaard S., Coupland S.E. (2016). Metastatic ocular melanoma to the liver exhibits infiltrative and nodular growth patterns. Hum. Pathol..

[25] Liao A., Mittal P., Lawson D.H., Yang J.J., Szalai E., Grossniklaus H.E. (2018). Radiologic and Histopathologic Correlation of Different Growth Patterns of Metastatic Uveal Melanoma to the Liver. Ophthalmology.

[26] Barnhill R., Vermeulen P., Daelemans S., Van Dam P.-J., Roman-Roman S., Servois V., Hurbain I., Gardrat S., Raposa G., Nicolas A. (2018). Replacement and desmoplastic histopathological growth patterns: A pilot study of prediction of outcome in patients with uveal melanoma liver metastases. J. Pathol. Clin. Res..

[27] Robertson A.G., Shih J., Yau C., Gibb E.A., Oba J., Mungall K.L., Hess J.M., Uzunangelov V., Walter V., Danilova L. (2018). Integrative Analysis Identifies Four Molecular and Clinical Subsets in Uveal Melanoma. Cancer Cell.

[28] Yu H., Pak H., Hammond-Martel I., Ghram M., Rodrigue A., Daou S., Barbour H., Corbeil L., Hébert J., Drobetsky E. (2013). Tumor suppressor and deubiquitinase BAP1 promotes DNA double-strand break repair. Proc. Natl. Acad. Sci. USA.

[29] Nguyen J.Q., Drabarek W., Yavuzyigitoglu S., Salsench E.M., Verdijk R.M., Naus N.C., De Klein A., Kiliç E., Brosens E. (2020). Spliceosome Mutations in Uveal Melanoma. Int. J. Mol. Sci..

[30] Dayani P.N., Gould J.E., Brown D.B., Sharma K.V., Linette G.P., Harbour J.W. (2009). Hepatic metastasis from uveal melanoma: Angiographic pattern predictive of survival after hepatic arterial chemoembolization. Arch. Ophthalmol..

[31] Bosch T.V.D., Van Beek J.G.M., Vaarwater J., Verdijk R.M., Naus N.C., Paridaens D., De Klein A., Kiliç E. (2012). Higher Percentage of FISH-Determined Monosomy 3 and 8q Amplification in Uveal Melanoma Cells relate to Poor Patient Prognosis. Investig. Opthalmol. Vis. Sci..

[32] Onken M., Worley L.A., Harbour J.W. (2008). A Metastasis Modifier Locus on Human Chromosome 8p in Uveal Melanoma Identified by Integrative Genomic Analysis. Clin. Cancer Res..

[33] Halenda K.M., Kudchadkar R.R., Lawson D.H., Kies D.D., Zhelnin K.E., Krasinskas A.M., Grossniklaus H.E. (2015). Reduction of Nodular Growth Pattern of Metastatic Uveal Melanoma after Radioembolization of Hepatic Metastases. Ocul. Oncol. Pathol..

[34] Field M.G., Decatur C.L., Kurtenbach S., Gezgin G., Van Der Velden P.A., Jager M.J., Kozak K.N., Harbour J.W. (2016). PRAME as an Independent Biomarker for Metastasis in Uveal Melanoma. Clin. Cancer Res..

[35] Babchia N., Landreville S., Clément B., Coulouarn C., Mouriaux F. (2019). The bidirectional crosstalk between metastatic uveal melanoma cells and hepatic stellate cells engenders an inflammatory microenvironment. Exp. Eye Res..

[36] Sharma K.V., Gould J.E., Harbour J.W., Linette G.P., Pilgram T.K., Dayani P.N., Brown D.B. (2008). Hepatic Arterial Chemoembolization for Management of Metastatic Melanoma. Am. J. Roentgenol..

[37] Valpione S., Moser J.C., Parrozzani R., Bazzi M., Mansfield A.S., Mocellin S., Pigozzo J., Midena E., Markovic S.N., Aliberti C. (2015). Development and External Validation of a Prognostic Nomogram for Metastatic Uveal Melanoma. PLoS ONE.

