# Supplementary material for: Radiological Patterns of Uveal Melanoma Liver Metastases in Correlation to Genetic Status
Source: Cancers (Basel). 2021 Oct 22;13(21):5316. doi: 10.3390/cancers13215316 (PMC8582397; doi:10.3390/cancers13215316)
Supplement: Supplementary file 1 [file cancers-13-05316-s001.zip › cancers-1437227-supplementary.pdf]

Radiological Patterns of Uveal Melanoma Liver Metastases in Correlation to Genetic Status

S. Yavuzyigitoglu, M. C. Y. Tang, M. Jansen, K. W. Geul, R. S. Dwarkasing, J. Vaarwater, W. Drabarek, R. M. Verdijk, D. Paridaens, N. C. Naus, E. Brosens, A. de Klein and E. Kilic

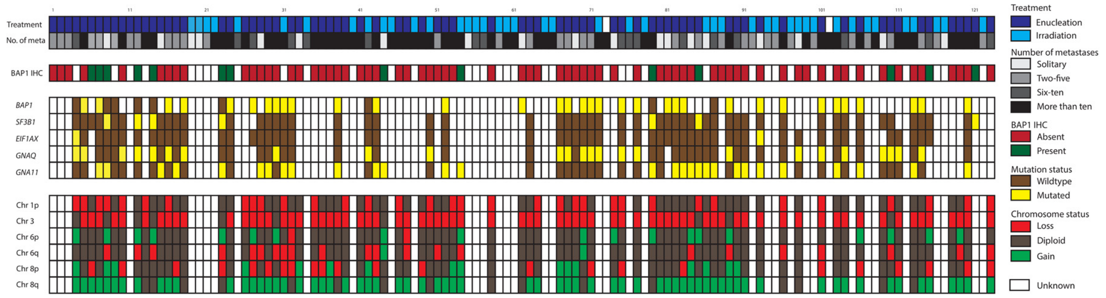

Figure S1. A schematic overview of all patients with UM with the subsequent treatment, number of metastases, BAP1 IHC, mutations in *BAP1*/*SF3B1*/*EIF1AX*/*GNAQ*/*GNA11*, and abnormalities of chromosome 1p/3/6p/6q/8p/8q.
